# Potentials of leaves of *Aspilia africana *(Compositae) in wound care: an experimental evaluation

**DOI:** 10.1186/1472-6882-7-24

**Published:** 2007-07-10

**Authors:** CO Okoli, PA Akah, AS Okoli

**Affiliations:** 1 Department of Pharmacology & Toxicology, Faculty of Pharmaceutical Sciences, University of Nigeria, Nsukka 410001, Enugu State, Nigeria; 2 Department of Microbiology, University of Nigeria, Nsukka 410001, Enugu State, Nigeria; 3 Department of Pathology, School of Medical Sciences, Faculty of Medicine, University of New South Wales, Sydney, 2052, Australia

## Abstract

**Background:**

The potentials of the leaves of the haemorrhage plant, *Aspilia africana *C. D Adams (Compositae) in wound care was evaluated using experimental models. *A. africana*, which is widespread in Africa, is used in traditional medicine to stop bleeding from wounds, clean the surfaces of sores, in the treatment of rheumatic pains, bee and scorpion stings and for removal of opacities and foreign bodies from the eyes. The present study was undertaken to evaluate the potentials for use of leaves of this plant in wound care.

**Methods:**

The effect of the methanol extract (ME) and the hexane (HF) and methanol (MF) fractions (obtained by cold maceration and graded solvent extraction respectively) on bleeding/clotting time of fresh experimentally-induced wounds in rats, coagulation time of whole rat blood, growth of microbial wound contaminants and rate of healing of experimentally-induced wounds in rats were studied as well as the acute toxicity and lethality (LD_50_) of the methanol extract and phytochemical analysis of the extract and fractions.

**Results:**

The extract and fractions significantly (*P *< 0.05) reduced bleeding/clotting time in rats and decreased coagulation time of whole rat blood in order of magnitude of effect: MF>ME>HF. Also, the extract and fractions caused varying degrees of inhibition of the growth of clinical isolates of *Pseudomonas fluorescens *and *Staphylococcus aureus*, as well as typed strains of *Ps. aeruginosa *(ATCC 10145) and *Staph. aureus *(ATCC 12600), and reduced epithelialisation period of wounds experimentally-induced in rats. Acute toxicity and lethality (LD_50_) test in mice established an i.p LD_50 _of 894 mg/kg for the methanol extract (ME). Phytochemical analysis revealed the presence of alkaloids, saponins, tannins, flavonoids, resins, sterols, terpenoids and carbohydrates.

**Conclusion:**

The leaves of *A. africana *possess constituents capable of arresting wound bleeding, inhibiting the growth of microbial wound contaminants and accelerating wound healing which suggest good potentials for use in wound care.

## Background

Wounds occur when the continuity of the skin or mucous membrane is broken [1]. Injury to tissues results in bleeding (which may be life-threatening depending on the severity) with subsequent activation of acute inflammatory reactions. Bleeding from damaged blood vessels in the injured tissue must be arrested through the process of haemostasis. The injury and associated acute inflammatory response result to necrosis of specialized cells and damage to the surrounding matrix [2] and the host tissues must activate the healing process to replace dead tissues with healthy ones. However, microbial infection of the wound impairs the healing process [2] and may delay tissue repair. Besides the pain and general discomfort arising from injuries or wounds there may be alteration of function which interferes with the individual's ability to carry on with daily life activities. Consequently, there is an overriding need to stimulate healing and restore the normal functions of the affected part(s) of the body to ease the discomfort and pain associated with wounds by arresting bleeding from fresh wounds, preventing infection, and activating tissue repair processes.

Several medicinal plants are used in folklore for wound treatment. One of such plants is *Aspilia africana *C.D. Adams (Compositae), a herb about 1 m tall covered with bristles [3] and commonly known as "haemorrhage plant" due to its ability to stop bleeding from fresh wounds [4]. In Nigeria, it is variously known as "Orangila" in Igbo, "Tozalin" in Hausa, "Yunyun" in Yoruba and "Edemedong" in Efik [5] and widespread in Africa [6]. The morphological features have been fully described [3,6]. In wound care in herbal medicine, the bruised leaves and flowers of *A. africana *are used to clean the surfaces of sores [5] with subsequent healing [7]. In addition, it is also used for the treatment of rheumatic pains [4] as well as bee and scorpion stings [5]. The decoction has been used to remove corneal opacities and foreign bodies from the eyes [5]. The haemostatic [8], antibacterial [9], membrane stabilization [10] and anti-inflammatory [11] activities of *A. africana *have been reported. The leaf extract has also been shown to cause extracellular Ca^2+ ^dependent increase in vascular tone [12]. The isolation of a number of terpenoids from the leaves of *A. Africana *has been documented. Sesquiterpenes and monoterpenes were isolated from the essential oil of the leaves [13]. Germacrene D was reported as the major sesquiterpene while α-pinene and limonene were the major monoterpenes [13]. A comparative phytochemical analysis of the leaves of *A. africana *has identified saponins and tannins as the most abundant constituents of the plant [14].

The ethnomedicinal uses of this plant suggest its usefulness in wound treatment and stimulated our interest to study the leaf extracts for potential application in wound care.

## Methods

### Plant material

Fresh leaves of *A. africana *were collected in October 2003 within the premises of University of Nigeria, Nsukka and authenticated by Mr. A. Ozioko of the Bioresources Development and Conservation Program (BDCP) Center, Nsukka, Enugu State, Nigeria where a voucher specimen (BDCP 206) is maintained. The leaves were cut into smaller pieces, dried under the sun for 2 days and pulverized to powder using a hand blender.

The leaf powder (250 g) was extracted with methanol by cold maceration for 48 h to obtain the methanol extract (ME). A fresh batch of leaf powder (500 g) was successively extracted with n-hexane and methanol to obtain the n-hexane (HF) and methanol fractions (MF). The extract and fractions were concentrated in a rotary evaporator under reduced pressure to afford 10.2 g of methanol extract (ME), 2.1 g of n-hexane fraction (HF) and 17.8 g of methanol fraction (MF). The extract and fractions were subjected to phytochemical analysis for identification of constituents using standard methods [15].

### Pharmacological tests

#### Animals

Adult Swiss albino mice and rats and guinea pig of both sexes were obtained from the laboratory animal facility of the Department of Pharmacology & Toxicology, Faculty of Pharmaceutical Sciences, University of Nigeria, Nsukka. The animals were housed in groups in cages within the facility and maintained freely on standard pellets and water. They were allowed to acclimatize to the work area environment for two weeks prior to use. All animal experiments were in compliance with the National Institute of Health Guide for Care and Use of Laboratory Animal (Pub No. 85-23, revised 1985).

#### Acute toxicity and lethality (LD_50_) test

The acute toxicity and lethality (LD_50_) of the methanol extract (ME) was determined in mice using the method of Lorke [16]. Animals in groups of three received one of 10, 100, or 1000 mg/kg of ME suspended in 3% v/v Tween 85 administered intraperitoneally and observed for 24 h for number of deaths. From the results of the first test, 200, 400, 800, and 1600 mg/kg doses of the extract were administered to a fresh batch of animals at one animal per dose and the number of deaths in 24 h recorded. The LD_50 _was calculated as the geometric mean of the highest non-lethal dose (800 mg/kg) and the lowest lethal dose (1000 mg/kg).

#### Bleeding/clotting time test

The effect of the extract and fractions on bleeding from fresh experimentally-induced wounds was evaluated using the bleeding/clotting time test in rats. Briefly, adult albino rats (200–250 g) of both sexes were divided into five groups of three animals each. Each group received one of the ME, HF or MF suspended in 3% v/v Tween 85. Control animals received either the vehicle or normal saline. The tail of each rat was cut with a sharp pair of scissors. Immediately, a drop of the extract/fraction (100 μg/ml) was placed on the cut and at the same time a stopwatch was started. The cut was dabbed with a small piece of filter paper every 15 s until the paper no longer stained red with blood oozing from the cut. Bleeding time was taken as the time for the first drop of blood to show to the time when the filter paper stopped showing bloodstain [17].

#### Coagulation time of whole blood

The effect of the extract and fractions on coagulation of fresh blood was evaluated using the coagulation time of whole rat blood. Adult albino rats (200–250 g) of both sexes were divided into 8 groups of three animals each. Each animal was anaesthetized with chloroform and the thoracic cavity opened to expose the aorta. The aorta was severed and 1 ml of blood withdrawn using a plastic disposable syringe. The blood was quickly delivered into clean paraffin-coated glass tubes (10 mm diameter) containing 0.5 ml of one of the ME, HF or MF (10, 100 ug/ml) suspended in 3% v/v Tween 85. The vehicle or normal saline was used as the control. The glass tubes were swirled every 15 s to check the fluidity of the contents. The interval between the introduction of the blood and the time of clot formation was taken as the coagulation time [17].

#### Antimicrobial activity test

The effect of extract and fractions on microbial wound contaminants was evaluated using the antimicrobial activity on wound isolates. Clinical wound isolates were collected in sterile swab sticks from patients prior to dressing of the wounds. These patients were selected without age and gender discrimination and all had infected wounds from various causes. The swabs were streaked and subcultured three times in sterile nutrient agar plates and subsequently maintained on agar slants stored at 4°C. The isolates were characterized and identified using gram staining, colony characterization, cetrimide agar, gelatin liquefaction, sodium chloride and mannitol fermentation tests [18]. Antimicrobial activity test was performed using the agar well diffusion method of Lovian [19]. Briefly, sterile Muller Hinton agar plates were flooded with 1 × 10^6 ^cfu/ml concentration of microorganisms. Using a sterile cork borer (7 mm diameter), 6 wells were bored on the agar and three drops of one of the ME, HF or MF (0.5, 0.25, 0.125, 0.0625, 0.03125 mg/ml in 10% dimethylsulfoxide (DMSO) placed in the appropriate well. DMSO (10%) was used as control. The plates were allowed 30 min for diffusion and incubated inverted for 24 h at 37°C. Microbial sensitivity was determined in triplicate. After incubation, the diameter of inhibition zone for each well was measured horizontally and vertically and the mean obtained. The minimum inhibitory concentration (MIC) was determined as intercept on the concentration axis of Concentration vs mean IZD^2 ^plot.

#### Wound epithelialisation time test

The effect of the extract and fractions on the rate of wound healing activity was evaluated using wound epithelialisation time in rats. Adult albino rats (200–280 g) of both sexes were divided into 4 groups of 6 animals each. Each group received either 200 or 400 mg/kg of one of the ME, HF or MF suspended in 3% v/v Tween 85. Control animals received the vehicle. Each animal was anaesthetized with subcutaneous injection of lignocaine (2% w/v adrenaline 1:10 000). An excision wound of about 100 mm^2 ^was created on the inner surface of the right hind leg using sterile surgical blades. Extract and fractions were administered orally to the animals once daily for ten days starting from the day of wound creation. Wound circumference was measured daily for the ten days and the period of epithelialisation was calculated as the number of days required for the scar to fall off leaving no raw wound [20].

### Statistical analysis

Results were analyzed using one way analysis of variance (ANOVA) and expressed as Mean ± SEM. Data was further subjected to LSD post hoc test and differences between means accepted significant at *P *< 0.05.

## Results

### Extraction and phytochemical analysis

The extraction process yielded 4.08% of the methanol extract (ME), 0.42% of n-hexane fraction (HF) and 3.56% of methanol fraction (MF) (Table 1). The methanol extract (ME) gave positive reactions for alkaloids, saponins, tannins, flavonoids, resins, steroids, terpenoids and carbohydrates. The n-hexane fraction (HF) tested positive for sterols and terpenoids while the methanol fraction (MF) gave positive reactions for alkaloids, glycosides, saponins, flavonoids, tannins, resins, steroids and carbohydrates (Table 1).

**Table 1:** Phytochemical constituents of extract and fractions

**Phytochemical constituents**	**Extract and fractions**		
	
	**ME (4.08%)**	**HF (0.42%)**	**MF (3.56%)**
Alkaloids	+	-	+
Glycosides	-	-	+
Saponins	+	-	+
Tannins	+	-	+
Flavonoids	+	-	+
Resins	+	-	+
Sterols	+	+	-
Terpenoids	+	+	-
Carbohydrates	+	-	+

Values in parenthesis are extractive yields. + = Present; - = Absent.

ME = Methanol extract; HF = n-Hexane fraction; MF = Methanol fraction

### Acute toxicity and lethality (LD_50_) test

The acute toxicity testing of the methanol extract (ME) in mice gave an i.p LD_50 _of 894 mg/kg (Table 2).

**Table 2:** Result of acute toxicity and lethality (LD_50_) test

**Stage of test**	**Dose (mg/kg)**	**Number of death per group**
I (Determination of toxic range of extract)		
	10	0/3
	100	0/3
	1000	2/3
II (Determination of lethality)		
	200	0/1
	400	0/1
	800	0/1
	1600	1/1

The i.p LD_50 _was calculated as 894 mg/kg.

### Effect of extract and fractions on bleeding/clotting time and coagulation time of whole blood

The extract and fractions significantly (*P *< 0.05) reduced bleeding/clotting time in rats. On coagulation of whole blood, the extract and fractions decreased the coagulation time of whole rat blood in a dose-dependent manner. In both tests, the magnitude of activity is of the order: MF>ME>HF (Table 3).

**Table 3:** Bleeding/clotting and whole rat blood coagulation time

**Extract/fraction**	**Concentration (μg/ml)**	**Bleeding/clotting time (s)**	**Coagulation time (s)**
ME	10	NT	102.67 ± 31.08
	100	31.33 ± 0.67^a, b^	55.67 ± 8.95
			
HF	10	NT	115.00 ± 26.50
	100	42.00 ± 2.31^a, b^	64.00 ± 25.53
			
MF	10	NT	98.33 ± 36.84
	100	30.33 ± 3.84^a, b^	42.67 ± 2.40^a^
			
Normal saline		54.00 ± 2.18	48.67 ± 5.20
Control		52.00 ± 0.58	89.33 ± 9.39

^a, b^*P *< 0.05 compared to Control and normal saline respectively (*ANOVA; LSD post hoc*); Values of bleeding and coagulation time shown are Mean ± SEM (n = 3); ME = Methanol extract; HF = n-Hexane fraction; MF = Methanol fraction; NT = Not tested.

### Effect of extract and fractions on microbial wound contaminants

Characterization of the clinical wound isolates established their identity as species of *Pseudomonas fluorescens *and *Staphyloccocus aureus *(Table 4). The extract and fractions exhibited varying levels of inhibitory effect on these bacteria. The methanol fraction (MF) caused the greatest inhibitory effect against all the organisms followed by ME with the n-hexane fraction (HF) exhibiting the least activity (Table 4).

**Table 4:** Minimum inhibitory concentration of extract and fractions

**Microorganism**	**Minimum inhibitory concentration (MIC) (mg/ml)**		
	
	**ME**	**HF**	**MF**
*Ps. fluorescens *(Isolate 1)	0.125	0.25	0.25
*Ps. fluorescens *(Isolate 2)	-	-	-
*Ps. fluorescens *(Isolate 3)	0.25	0.5	0.5
*Ps. fluorescens *(Isolate 4)	0.125	-	0.5
*Ps. fluorescens *(Isolate 5)	0.25	0.25	-
*Ps. fluorescens *(Isolate 6)	-	0.25	0.125
*Ps. fluorescens *(Isolate 7)	0.125	0.5	0.125
*Ps. aeruginosa (*ATCC 10145)	0.125	-	-
*Staph. aureus *(Isolate 1)	0.5	0.5	0.063
*Staph. aureus *(ATCC 12600)	-	-	0.25
Control	-	-	-

Control = 10% Dimethylsulfoxide (DMSO)

ME = Methanol extract; HF = n-Hexane fraction; MF = Methanol fraction - = No activity

### Effect of extract and fractions on wound epithelialisation time

The extract and fractions significantly (*P *< 0.05) reduced epithelialisation time in rats in a non-dose-related manner. The methanol fraction (MF) caused the highest reduction while ME and n-hexane fraction (HF) exhibited comparable levels of reduction (Table 5).

**Table 5:** Effect of extract and fractions on epithelialisation time in rats

**Extract/fractions**	**Dose (mg/kg)**	**Epithelialisation**	
		
		**Time (Days)**	**Reduction (%)**
ME	200	7.67 ± 0.33^a^	25.75
	400	8.33 ± 0.33^a^	19.36
			
HF	200	8.00 ± 0.00^a^	22.56
	400	8.30 ± 0.33^a^	19.65
			
MF	200	6.67 ± 0.67^a^	35.43
	400	7.67 ± 0.88^a^	25.75
			
Control	-	10.33 ± 0.67	-

^a ^*P *< 0.05 compared to Control (*ANOVA;LSD post hoc*); Values of epithelialisation time shown are Mean ± SEM (n = 3); ME = Methanol extract; HF = n-Hexane fraction; MF = Methanol fraction

## Discussion

Evaluation of the potentials of *A. africana *in wound care showed that the leaf extract and fractions exhibited haemostatic, antimicrobial and wound healing activities suggesting that the constituents of the leaves may play a useful role in wound care. The extract and fractions arrested bleeding from fresh wounds by reducing bleeding/clotting and whole blood coagulation time which are important indices of haemostatic activity. In separate studies elsewhere, *A. africana *leaf extracts demonstrated haemostatic activity [8,14]. Haemostasis involves the spontaneous arrest of bleeding from damaged blood vessels [21] which is important for initiation of tissue repair processes and prevention of tissue death through haemorrhage. The haemostatic process proceeds through a cascade of reactions, which starts with vascular spasm of the ruptured vessels [22,23], formation of platelet plug through platelet aggregation, and coagulation of the blood [22]. Leaf extracts of *A. africana *have been shown to increase vascular tone [12] which is a measure of vasoconstriction and suggestive of the possibility of the leaf extracts arresting bleeding from fresh wounds through this mechanism. However, blood clotting and coagulation also involve other mechanisms such as prothrombin activation with its subsequent conversion to thrombin and which in turn converts fibrinogen to insoluble fibrin [22]. The reduction of coagulation time of whole blood by the leaf extracts is an indication that the extracts may also interfere with the blood coagulation pathways. Thus, the haemostatic effect of the extract may derive from acceleration of the coagulation process with the consequent reduction in clotting time as well as vasoconstriction which are necessary in limiting blood loss from damaged vessels.

Wounds provide environments conducive for the growth of microbial organisms. Usually, microbial contaminations of wounds involve a variety of organisms such as *Pseudomonas aeruginosa, Staphylococcus aureus, Streptococcus faecalis, Escherichia coli, Clostridium perfringens, Clostridium tetani, Coliform *bacilli and enterococcus [24,25]. Evaluation of the effect of the extractives on clinically isolated microbial contaminants of wounds showed varying levels of inhibitory activity against species of *Pseudomonas *and *Staphylococcus*. Microbial infection of wounds delays healing [25,26] and causes a more pronounced acute inflammatory reaction [2] which can lead to further tissue injury and damage. Thus, the antimicrobial activity of the extract and fractions on these wound isolates may partly contribute to the wound healing effect by eliminating infection thus allowing the natural tissue repair processes to start. It also suggests that the leaf extracts may also play a useful role in accelerating the healing of old wounds by eradicating already established infection. The antimicrobial activity of honey and the essential oil of *Melaleuca alternifolia *is believed to underlie their usefulness as alternative therapy in wound healing [25,27,28].

In addition to inhibiting the growth of these micro-organisms, the extract and fractions effectively reduced the epithelialisation period of experimentally-induced wounds which is an index of pro-healing activity. The precise aspect as well as the exact mechanism of wound healing affected by the extract and fractions is yet to be elucidated. In the tissue repair process, inflammatory cells promote the migration and proliferation of endothelial cells, leading to neovascularisation of connective tissue cells which synthesize extracellular matrices including collagen, and of keratinocytes resulting to re-epithelialisation of the wounded tissue [29]. In the wound healing process, collagen formation peaks at day 7 and epithelialisation occurs in 48 h under optimal conditions [30]. The extent to which the extractives interact with these processes is not known to us.

Phytochemical analysis of the extract and fractions indicated the presence of typical plant constituents such as alkaloids, saponins, sterols, terpenoids, carbohydrates, glycosides and tannins. These metabolites are usually responsible for the pharmacological activities of medicinal plants. α-pinene, one of the terpenoids in *A. africana *leaves, is known to possess anti-inflammatory activity [31] and may contribute to the wound healing activity by suppressing inflammatory reactions invoked by the injured tissues. In addition to this, the documented identification of the abundant presence of saponins and tannins in the leaves of this plant [14] is implicating for these constituents in the activities of the leaf extracts, especially tannins, which have been implicated in the haemostatic activity of plants where they arrest bleeding from damaged or injured vessels by precipitating proteins to form vascular plugs. To a reasonable extent, going by the quantified relative presence in the leaves of this plant and documented role in haemostatic activity, we may safely assume that the tannins in the extracts partly contribute to the activity since mechanisms other than vascular plugs formation are likely involved.

## Conclusion

The leaf extract and fractions of *A. africana *effectively arrested bleeding from fresh wounds, inhibited microbial growth of known wound contaminants and accelerated wound healing process. The results of this study indicate that extracts of leaves of *A. africana *have good potentials for use in wound care and further provide a rationale for the use of the leaves of this plant in wound management in traditional medicine practice.

## Competing interests

The author(s) declare that they have no competing interests.

## Authors' contributions

COO designed the study, carried out the pharmacology experiments and statistical analysis and drafted the manuscript; PAA participated in the experimental design, coordination of experimental work and preparation of the manuscript; ASO carried out the antimicrobial experiments and participated in preparation of the manuscript. All authors read and approved the final manuscript.

## Pre-publication history

The pre-publication history for this paper can be accessed here:


